# Typhus Abdominalis

**Published:** 1843-08

**Authors:** 


*Typhus Abdominalis.*—Much new light has been lately thrown upon the pathology of this form of disease (typhus) by the researches and observations of Professor Rokitansky, and which have been made known to the profession in this country by my friends and colleagues in Vienna, Drs. Drysdale and Russell, in their valuable articles on the Pathology of Typhus, in the note to "Fletcher's Elements of General Pathology." From this we learn that "the Typhus process is characterized in an anatomical point of view by the deposition of a peculiar morbid product' which forthwith undergoes a distinct series of peculiar changes. The seat of this process is various, and depends upon the specific relation of the general process to certain organs. The tissues most subject to this deposition are the mucous membrane and the lymphatic glands; and in Austria, at least, where the observations were made, the mucous membrane of the ileum (*ilio-typhus*) is most frequently affected, but it also occurs in the bronchia and lungs, (when in this seat constituting most probably the *exanthematic typhus*), and also, though very rarely, in the colon (*colo-typhus*)."

Let us examine its changes in the intestines—the most frequent seat of the process. First, the stage of congestion, corresponding to the period of irritation and catarrhal or gastric symptoms. "It exhibits a congested state of the vessels, or succulent condition of the mucous membrane, particularly of the villous coat extending over the greater part of the ileum, but better marked at particular spots—especially near the cœcum; the mesenteric glands are slightly swollen, their vessels are gorged with blood, their substance soft and elastic, and their color dark.

"In the second stage (the stage of deposition of the morbid product, *i. e.* typhus infiltration, which is in respect to the degeneration its crude stage) the congested stage moderates to a certain degree, and is reduced to several spots corresponding to the Peyerian glands, and a few solitary follicles. Here it appears in the form of round elliptic '*plaques*,' varying from half a line to three lines in thickness, which are formed by the deposition of a peculiar substance in the Peyerian plexus and submucous cellular tissue. On a more close examination the degeneration is found to be so deposited in the submucous tissue of the follicles, that the deepest layer of that tissue, immediately covering the muscular coat, remains free from infiltration. It very seldom reaches beyond the bounds of the follicular apparatus.

"The mesenteric glands are now more swollen, so as to reach the size of a bean or hazel nut, blue, or grayish-red, tolerably consistent, and apparently infiltrated with lardaceous substance.

"The commencement of the the third stage (that of intumescence, softening nnd throwing off of the degeneration) is indicated by a return of the congestion in the ileum in a violent degree. The vessels, especially the veins, both in the mesentery and in their ramifications in the intestines, are gorged with dark-violet viscid blood, and the tissue of the mucous membrane presents again a swollen appearance, more especially in the villi, which yield a grayish-white turbid fluid on pressure.

"But the most remarkable change takes place in the *typhus-plaques* and mesenteric glands, which became spongy and turgescent. These changes may take place in either of the following ways. The deposit assumes the appearance of a grayish marrow-like substance, and is then, along with the covering of the mucous membrane adhering to it, converted into a dirty yellowish-brown eschar; which, shrinking together from all sides, gradually loosens itself at the margin, splits into various directions, breaks off from the deepest stratum of the submucous cellular tissue, and is carried off all at once, or by a repeated recurrence of the process; or the deposit degenerates into a loose, vascular, blood-streaked, bluish-red, luxuriant fungoid structure, which becomes a special source of profuse intestinal hemorrhages, and is generally thrown off piecemeal without scabbing.

5 *

"The mesenteric glands now reach their greatest volume, attaining the size of a pigeon's, and near the cœcal valve not unfrequently that of a hen's egg. Their substance is injected, tolerably consistent, but changes into a grayish-red, loose pulp, frequently presenting the appearance of evident extravasation of blood. It is then soft and elastic, or frequently gives rise to the sensation of fluctuation.

"In the fourth stage, after this deposition is thrown off, a loss of substance of the internal surface of the gut remains, which represents the proper typhus ulcer. It is unnecessary to go more into the detail or the appearance of these ulcers, which have been so frequently described and are so well known. At this stage the morbid product has been thrown off; the mesenteric glands diminish forthwith in volume by the removal of the infiltrated morbid matter, but they remain still somewhat larger than natural, and retain a reddish color and their increased vascularity.

"When the typhus process localizes itself in the bronchial mucous membrane, the phenomenon presents a considerable difference. It appears always in the form of a diffused intense congestion, with dark-colored swelling of the membrane, and copious secretion of a gelatinous, occasionally dark blood-streaked mucous, and is principally developed in the bronchial ramifications of the inferior lobe. It appears, therefore, in this situation tc be always arrested in the stage of typhus congestion, and never comes to any manifest production of that morbid product in the tissue of the bronchial mucous membrane, which is produced in such abundance in the follicular apparatus of the intestinal mucous membrane in abdominal typhus. Thus in the primitive *broncho-typhus* the general affection is localized in the bronchial mucous membrane alone, to the exclusion of all the other mucous membranes, even that of the intestines, with which the typhus process in general has the greatest affinity. In many cases indeed the latter exhibits a recognizable, but always subordinate secondary affection of the follicles, in which the neighboring mesenteric glands participate; and it would often be difficult to recognise the affection as typhus, were it not for the other attendant changes which mark the disease—viz: the peculiar engorgement of the spleen and congestion of the pyloric extremity of the stomach, the condition of the blood, and the typhus nature of the affection in general, but more especially the change in the bronchial glands. This change is the same as that which the mesenteric glands undergo in the *ili-typhus.*

They are swollen to the size of a pigeon's or hen's egg, reddish blue, spongy, friable, soft, and infiltrated with the peculiar typhus product.

"This form is frequently combined with *pneumo-typhus* and typhus pleurisy, and it undoubtedly the pathological cause of the exanthematic contagious typhus, and most probably also of the Irish and North American typhus, which commonly run their course without abdominal affection.

"Sometimes the typhus affection of the lungs is better pronounced than that of the bronchia; but the former never occurs independently of the latter.

"Besides these primary seats of typhus deposition it may be deposited in many other organs as a secondary formation, giving rise to many complications, which, though very interesting in a practical point of view, would lead to too much detail were we to notice them here.

"Third. The typhus matter has, even at its origin, but much more in the transformations it undergoes, the greatest analogy with the cancerous degeneration, and more particularly with the medullary cancer.

"Fourth. The local typhus process is an inflammation, not however of a healthy character, but of a typhus character; and this unhealthiness is given, according to Rokitansky, by the peculiar diseased state of the blood. Lastly, Rokitansky is of opinion that when the local process is not met with in the mucous membrane of the intestines or in any other mucous membranes, it may have run its course in the blood without localizing itself at all."—"*Rokitansky's Handbuch der Pathologischen Anatomie.*"

A perusal of the foregoing remarks shows that its post-mortem appearances resemble very closely the Parisian form of the disease. The Viennese pathologists, however, consider it a dyscratic affection, depending enterely upon the formation and subsequent changes of the peculiar substance called "typhus matter," similar in nature to medullary-sarcoma.

*Amer. Journ.*, from *Wilde's Austria.*

